# Production of Lipid Constructs by Design via Three-Dimensional Nanoprinting

**DOI:** 10.3390/mi14020372

**Published:** 2023-02-02

**Authors:** Yuqi Huang, Arpad Karsai, Pallavi D. Sambre, Wan-Chih Su, Roland Faller, Atul N. Parikh, Gang-yu Liu

**Affiliations:** 1 Department of Chemistry, University of California, Davis, CA 95616, USA; 2 Department of Materials Science and Engineering, University of California, Davis, CA 95616, USA; 3 Department of Biomedical Engineering, University of California, Davis, CA 95616, USA; 4 Department of Chemical Engineering, University of California, Davis, CA 95616, USA

**Keywords:** 3D nanoprinting, atomic force microscopy (AFM), lipid, 1-palmitoyl-2-oleoyl-sn-glycero-3-phosphocholine (POPC), organizational chiral structures

## Abstract

Atomic force microscopy (AFM) in conjunction with microfluidic delivery was utilized to produce three-dimensional (3D) lipid structures following a custom design. While AFM is well-known for its spatial precision in imaging and 2D nanolithography, the development of AFM-based nanotechnology into 3D nanoprinting requires overcoming the technical challenges of controlling material delivery and interlayer registry. This work demonstrates the concept of 3D nanoprinting of amphiphilic molecules such as 1-palmitoyl-2-oleoyl-sn-glycero-3-phosphocholine (POPC). Various formulations of POPC solutions were tested to achieve point, line, and layer-by-layer material delivery. The produced structures include nanometer-thick disks, long linear spherical caps, stacking grids, and organizational chiral architectures. The POPC molecules formed stacking bilayers in these constructions, as revealed by high-resolution structural characterizations. The 3D printing reached nanometer spatial precision over a range of 0.5 mm. The outcomes reveal the promising potential of our designed technology and methodology in the production of 3D structures from nanometer to continuum, opening opportunities in biomaterial sciences and engineering, such as in the production of 3D nanodevices, chiral nanosensors, and scaffolds for tissue engineering and regeneration.

## 1. Introduction

Lipid molecules are important biological materials as the main building blocks for cellular membranes, and for engineered materials, biomimetic systems, and drug delivery carriers. Lipid constructs are well-tolerated by the human body with high stability and biodegradability, and the ability to carry hydro- and lipophilic compounds [1,2]. Microengineered 2D and 3D lipid assemblies were widely reported in cellular studies, and as biomimetic systems and drug delivery carriers [3,4,5,6,7]. The production of lipid structures is most commonly carried out via drop-casting [8], Langmuir–Blodgett (LB) deposition [9,10], Langmuir–Schaefer (LS) transfer [11], and vesicle fusion (VS)-based technologies [12]. While simple to operate, the mixtures of lipid assemblies with broad variations are generated. Designed two-dimensional lipid nanostructures could be probed via AFM-based nanolithography [13,14], but ut is difficult to extend probing to 3D due to difficulties in controlling interlayer registry. While there have been attempts to macroscale 3D-print [15] and nanoscale 2D-pattern lipids [5], the 3D nanoprinting of lipids remains challenging [16]. On the other hand, 3D printing, e.g., direct writing, enabled the production of the designed structures [17] of various materials, including polymers [18,19], polysaccharides [20], hydrogels [21,22,23], and proteins [24,25]. Three-dimensional printing and stereolithography [26,27,28] enabled the production of the design structures of photopolymers. Despite success with micro- and macroscales, further miniaturization for the production of 3D nanoarchitectures of lipids remains very challenging [29,30,31].

This work introduces a new means to address these challenges that combines the high spatial precision of atomic force microscopy (AFM) and the nanodelivery capability of microfluidic probes, which enables the production of the 3D nanostructures of polymers and carbohydrates [4,19,32,33,34,35,36]. This work tests its applications using amphiphilic lipid molecules. Various formulations of 1-palmitoyl-2-oleoyl-sn-glycero-3-phosphocholine (POPC) solutions were tested to reach the designed material delivery. Various lipid structures were designed and produced, including nanometer-thick disks, long linear spherical caps, stacking grids, and organizational chiral structures. Our high-resolution structural characterization reveals the spatial precision of the 3D nanoprinting in nanometers over the range of 0.5 mm. The results demonstrate the feasibility and concept of producing the 3D structures of lipids from a nanometer to continuum, opening opportunities in biomaterial sciences and engineering such as the production of 3D nanodevices, chiral nanosensors, and scaffolds for tissue engineering and regeneration.

## 2. Materials and Methods

### 2.1. Materials and Supplies

Glass slides and glass coverslips were purchased from Fisher Scientific (Pittsburgh, PA). Glycerol (>99%), sulfuric acid (H_2_SO_4_, 95.0–98.0%), hydrogen peroxide (H_2_O_2_, 30% aqueous solution), ammonium hydroxide (NH_4_OH, 28.0–30.0% aqueous solution), and chloroform (99.8%) were purchased from Sigma-Aldrich (St. Louis, MO, USA). Octadecyltrichlorosilane (OTS) was purchased from Gelest (Morrisville, PA, USA). Ethanol (200 Proof pure ethanol) was purchased from Koptec (King of Prussia, PA, USA). Reagents were used without further purification. Milli-Q water (MQ water, 18.2 MΩ·cm at 25 °C) was produced with a Milli-Q water purification system (EMD Millipore, Billerica, MA, USA). Nitrogen gas (99.999%) was purchased from Praxair, Inc. (Danbury, CT, King of Prussia, PA, USA). 1-Palmitoyl-2-oleoyl-sn-glycero-3-phosphocholine (POPC) and 1,2-dioleoyl-sn-glycero-3-phosphoethanolamine-N-(7-nitro-2-1,3-benzoxadiazol-4-yl) (NBD-PE) were purchased from Avanti Lipids, Inc. (Alabaster, AL, USA).

### 2.2. Preparation of Glass Supports

Coverslips were first cleaned with ethanol and water, and plasma-cleaned for 5 min with a plasma cleaner (PDC-32G, Harrick Plasma, Ithaca, NY, USA). To prepare the OTS/glass coverslips, the glass substrates were first cleaned following the established protocols [33,37,38,39]. Briefly, the coverslips were first cleaned in piranha solution for 1 h. The piranha solution was prepared by mixing H_2_SO_4_ and H_2_O_2_ (*v*/*v* = 3:1). Then, the coverslips were rinsed with copious MQ water. The coverslips were then soaked in a basic bath that contained NH_4_OH, H_2_O_2_, and MQ water at a ratio of 5:1:1 (*v*/*v*) for 1 h at 70 °C. The clean glass coverslips were then rinsed with copious amounts of MQ water and dried in nitrogen gas. The OTS/glass coverslips were prepared shortly before using. Briefly, the clean glass coverslips were placed into a sealed Teflon container (100 mL) containing 225 µL of OTS and heated in an oven at 70 °C for 2 h. The modified coverslips were rinsed with ethanol and dried in nitrogen gas. This protocol yields a self-assembly monolayer of OTS on silica surfaces, including glass and silicon wafers [37,38,39,40].

### 2.3. Solution Contact Angle and Viscosity Measurements

Contact angle data were collected for the modified glass supports using a VCA Optima Contact Angle Measurement system (AST Products, Billerica, MA, USA) according to previously established protocols [33,37,41,42,43]. A 3 µL drop of the designated solvent was placed on glass supports using a Hamilton 700 series HPLC needle (Hamilton CO., Reno, NV, USA). At least three different locations per sample were tested to confirm the surface wettability. Viscosity measurement was taken using an Ostwald viscometer with a capillary diameter of 1.0 mm according to previously established protocols [44]. Pure ethanol was used as a reference, and the transit time taken for the designated solvent to pass the two premarked lines was measured. It was assumed that the solute had negligible contribution to the viscosity of the solution given the low molar ratio (0.0019) and the low molecular weight of the solute [45,46].

### 2.4. Three-Dimensional Nanoprinting Using Combined Atomic Force Microscopy and Microfluidic Delivery

**Figure 1:**
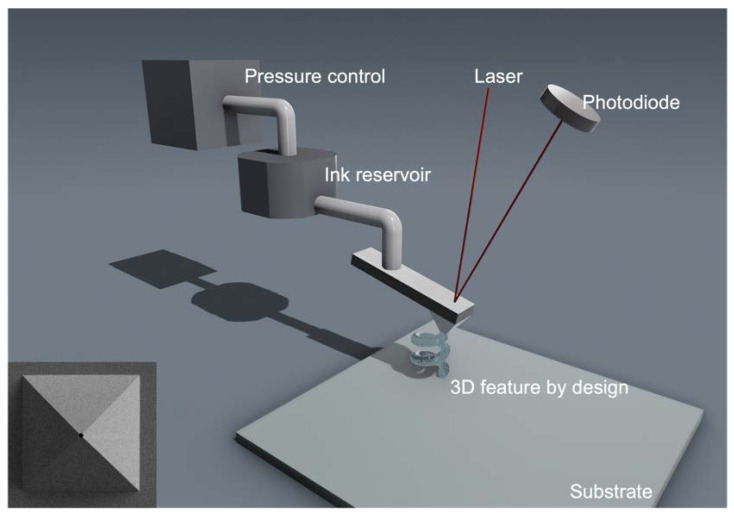
Schematic (not to scale) diagram of an AFM with microfluidic delivery probe. (inset) SEM image of the nanopipette apex with a 300 nm aperture.

The delivery process was carried out by using AFM-based technology in conjunction with a microfluidic delivery probe (FluidFM BOT, Cytosurge, Glattbrugg, Switzerland) [36,47]. Key components included an inverted optical microscope (IX-73, Olympus America, Center Valley, PA, USA), a nanopipette (Cytosurge, Glattbrugg, Switzerland) with a 300 nm opening connected to the material reservoir, as shown in Figure 1, control units for the material delivery (pressure and contact time), and for positioning and AFM probe contact [4,19,48,49,50]. The exteriors of the probes were coated with OTS before the actual printing process. Briefly, the probes were first cleaned with Milli-Q water and ethanol, and then dried in nitrogen gas. The clean probes were placed into a sealed Teflon container (100 mL) containing 150 µL of OTS and heated in an oven at 70 °C for 1 h. This protocol yields a self-assembly monolayer of OTS on silica surfaces, including glass and silicon wafers [37,38,39,40]. To prepare the lipid stock solutions, POPC was first dissolved in chloroform to produce a stock solution of 25 mg/mL. The NBD-PE stock solution was produced by dissolving NBD-PE in chloroform at a concentration of 5 mg/mL. The POPC/NBD-PE mixtures were prepared by mixing 39.5 µL of POPC stock solution and 2.5 µL NBD-PE solution, and were then allowed to dry under a stream of nitrogen gas. These stock solutions and mixtures were then used in POPC formulation for each 3D nanoprinting experiment discussed under Section 3.

### 2.5. Characterization of Supported Lipid Constructs Using Atomic Force Microscopy

The printed POPC constructs were cured and then imaged using an AFM (MFP-3D, Oxford Instrument, Santa Barbara, CA, USA). Silicon nitride probes (MSNL-10 E, Bruker, Camarillo, CA, USA) were used to characterize the geometry and size of the printed structures. Image acquisition was conducted using tapping mode with 60% damping [37,51]. Imaging processing and display were performed using MFP-3D software developed on the Igor Pro 6.20 platform.

### 2.6. Laser Scanning Confocal Microscopy Imaging

A laser scanning confocal microscope (FV-1000, Olympus America, Center Valley, PA, USA) was utilized for imaging the lipid patterns. A 10X bright field objective was used for visualizing the millimeter-scale patterned regions. Argon (458 nm) excitation and a 535–635 nm emission window were utilized for the NBD-PE signal [52]. The images were taken at an 8 µs/pixel scanning speed. Imaging processing and display were performed using FV10-ASW Viewer software (Ver.4.2b).

## 3. Results and Discussion

### 3.1. Production of Stacks of Lipid Bilayers

**Figure 2:**
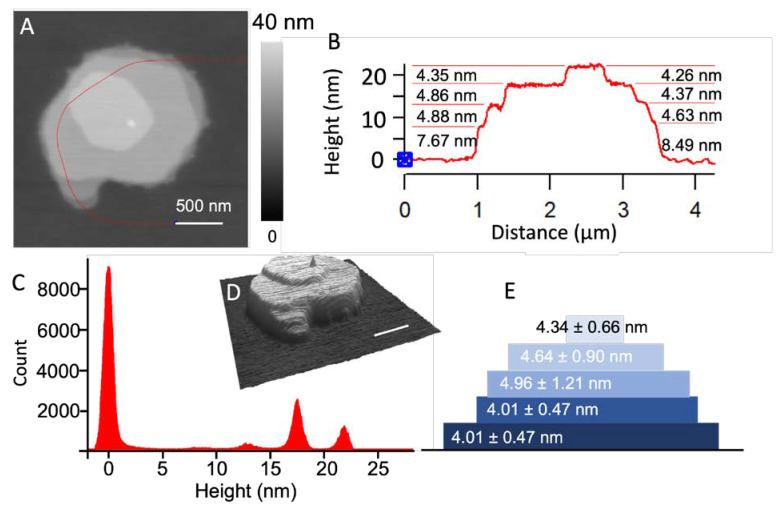
(**A**) AFM topographic image of a POPC feature formed during single-drop delivery. (**B**) Cursor profile indicated by the red line in (**A**). (**C**) Histogram of the topographic height measurements in (**A**). (**D**) A 3D display of (**A**). (**E**) Schematic diagram of the lipid structure produced in (**A**), i.e., five POPC bilayers stacking atop each other.

Figure 2A shows a representative POPC feature produced via single-point delivery on a clean glass surface, and each droplet was allowed to dry under ambient laboratory conditions. The lipid solution was produced by mixing ethanol:glycerol = 9:1 (*v*:*v*) and the POPC/NBD-PE mixture into a final concentration of 0.033 M POPC. The formulation of the ink is an important aspect of this work because the formulation significantly impacts the delivery outcomes, including the shape of the droplet, and the drying and printing of each droplet. To facilitate the formation of stacking POPC bilayers, we included 10% glycerol to the base lipid, dye, and ethanol mixture to slow down evaporation, allowing for the POPC molecules to assemble into bilayers. The measured viscosity of the solvent was 1.81 mPa·s at room temperature. A plasma-cleaned glass coverslip was used as support. Delivery was carried out under delivery pressure of 300 mbar and for a contact duration of 500 ms. High-resolution AFM imaging was carried out 42 days after delivery. In our experimental setting, the delivery of a 3 × 3 array of these droplets was completed within 10 s. A typical structure of these POPC from this protocol is shown in Figure 2A. The overall morphology exhibited a disk shape and 4 steps viewed from the edges, which is consistent with the stacking of layered materials, with each layer being polygonal (Figure 2A,D). The characteristic angles for the top lipid polygon were measured by extrapolating each edge and measuring the angle at the crossing point. The individual angles were 119.8 ± 2.9°, 105.2 ± 2.8°, 126.4 ± 3.1°, 119.4 ± 3.4°, 103.1 ± 2.1°, and 139.6 ± 3.1°. The measured angles were close to 120°, which is the characteristic angle for a closely packed 2D symmetry. The layer thickness was measured from the cursor profile, as indicated on the AFM topographic image in Figure 2B. Figure 2C shows the histogram analysis to show the statistic. From the bottom to the top, the layer thickness was 8.02 ± 0.94, 4.96 ± 1.21, 4.64 ± 0.90, and 4.34 ± 0.66 nm. The interbilayer distance for the macroscopic lipid stacks prepared in a drop-and-dry casting was 4.51 nm [8,53]. Our quantification of step height allowed for us to infer that the first layer likely consisted of five lipid bilayers, with the bottom portion containing two bilayers, while the remaining were single-lipid bilayers, as illustrated in Figure 2E. The results were reproducible within the experiment shown in Figure 2, where two arrays, 2 × 2 and 3 × 3, were produced.

### 3.2. Productions of Arrays of Lipid Cylindrical Caps

**Figure 3:**
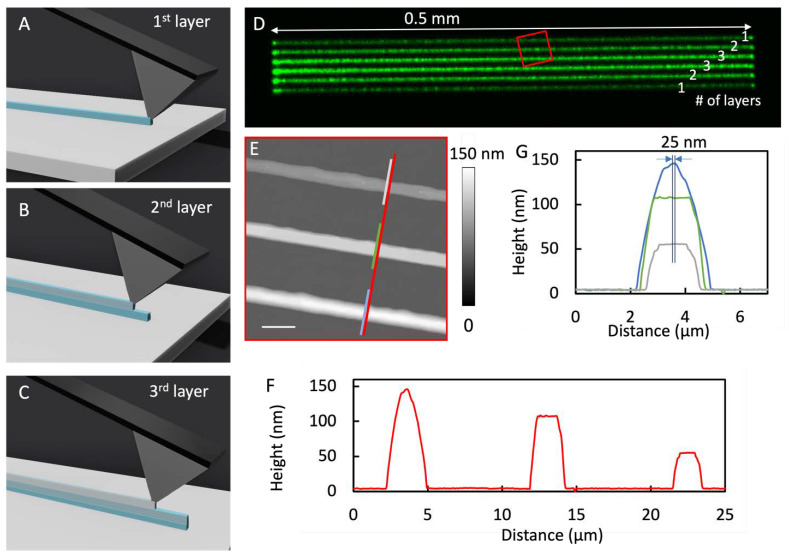
(**A**) Schematic diagram of a cylindrical cap printed with one pass of delivery. (**B**) Schematic diagram of the outcome of a second-pass delivery on top of the first. (**C**) Schematic diagram of the outcome of a third-pass delivery on top of the second. (**D**) Confocal image covering the 0.5 mm × 50 µm feature area that contained all 6 cylindrical caps. The number of passes is indicated on the right. (**E**) Topographic AFM image indicated by the red square in (**D**); scale bar = 5 µm. (**F**) Cursor profile as indicated by the red line in (**E**). (**G**) A combined view of the three cursor plots indicated in (**F**) to measure the interlayer registry, i.e., 25 nm precision.

To print arrays of line features, one could consider the process equivalent to printing connected droplets along the linear trajectory. To ensure continuity and homogeneity along the line, the ink formulation, spread property, and printing speed (i.e., transient contact time) needed to be tested to avoid broken lines or smudging. The lipid solution was produced by dissolving POPC at 0.033 M in ethanol. To facilitate structures with a high aspect ratio, e.g., cylindrical instead of pancake shapes, only pure ethanol was used, as the solvent to control spreading and accelerate acceleration in contrast to solvent containing glycerol. The final formulation had a solvent viscosity of 1.040 mPa·s at 25 °C. To visualize the patterns under a laser scanning confocal microscope, 1 mol % NBD-PE was added to the mixture as the fluorophore. The supports were OTS/glass surfaces. To produce the linear features with the shape of a cylindrical cap, the probe moved along a designated linear trajectory as shown in Figure 3A. Delivery was synchronized with the linear movement of the probe under delivery pressure of 10 mbar and with printing speed of 150 µm/s. The height of the feature could be adjusted via 3D printing, i.e., by overlaying the second pass of the delivery on top of the first, while the delivery conditions were kept the same. The procedure of the second and third passes is illustrated in Figure 3B,C, respectively. Laser scanning confocal imaging and high-resolution AFM imaging were performed 9 days after printing to ensure curing. The results of a typical set of experiments are shown in Figure 3D, where all six cylindrical caps are clearly visible, and interline separation measures at 10 µm. The fluorescence intensity of each observed linear feature (Figure 3D) is consistent with the number of passes (labeled on Figure 3D) utilized to generate each feature. The height of these features was measured from the topographic AFM image and cursor profile, as illustrated in Figure 3E,F, respectively. The heights of the cylindrical caps increased with the increasing number of passes: 52, 104, and 142 nm for the single, double, and triple passes, respectively. In this case, the third pass of delivery led to fewer materials than the first two passes did. This observation was likely due to a small reduction in aperture size after the first two passes, as some materials could become dry near the apex of the probe. Depending on the locations of these materials with respect to the aperture, a partial blockade could occur. The widths of the cylindrical caps, characterized with full width at half-maximum (FWHM), were 1.81 ± 0.11, 2.01 ± 0.04, and 1.93 ± 0.07 µm. The observation of a similar width and linear height increase with the number of passes demonstrates the high spatial precision of this nanoprinting procedure. The alignment of the second and third lines was within 25 nm (see Figure 3G) of the first pass over a 0.5 mm range.

Under the same design, we could easily tune the height of the cylindrical caps by varying the probe’s moving speed. For example, by decreasing printing speed from 150 to 8 µm/s, the height increased to 298, 453, and 678 nm for the cylindrical caps produced with single-, double-, and triple-pass delivery, respectively, while the widths remained constant (1.9–2.1 µm). These results successfully demonstrate the ability to produce line features via 3D nanoprinting design with nanometer precision over millimeter footprint.

### 3.3. Production of 3D Stacking Grids

**Figure 4:**
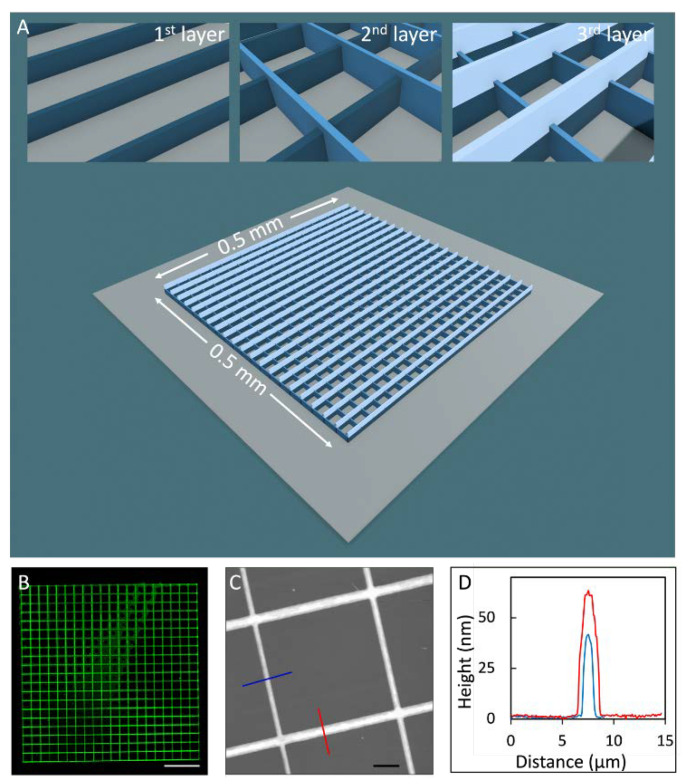
(**A**) Schematic diagram of the designed stacking grids of lipid molecules; (insert) the first, second, and third layers of the designed structures. (**B**) Confocal image revealing the 0.5 × 0.5 mm area containing the stacking grids. Scale bar = 100 µm. (**C**) Topographic AFM image providing high-resolution 3D information of the lipid grids. Scale bar = 5 µm. (**D**) A combined cursor profile from the two cursors in (**C**), where blue and red cursors cross the single- and double-layer grids, respectively.

With the successful ink formulation and printing conditions established as discussed in previous section, more complex designs were attempted, e.g., 3-layer stacking grids. The overall dimensions of the grids covered 0.5 × 0.5 mm, as illustrated in Figure 4A, within which 21 grids were parallel with periodicity of 25 µm. The second grid layer had the same design and was positioned perpendicular to the first and third layers (Figure 4B,C). Laser scanning confocal and high-resolution AFM images were taken 3 days after delivery to allow for the lipid constructs to cure.

As shown in Figure 4D, the outcome exhibited high fidelity with the design. A zoomed-in view is shown in the topographic AFM image in Figure 4C, revealing a typical grid. The widths of the single- and double-layered grids, characterized from the cursor profile (FWHM), measured 1.0 ± 0.1 and 1.7 ± 0.1 µm, respectively, as shown in Figure 4C. The heights were 46.3 and 61.1 nm for the single- and double-layered grids, respectively. Histogram analysis was also carried out on multiple randomly selected areas (0.5 × 0.5 mm) of which the single- and double- layered heights were 45.2 ± 1.2 and 61.6 ± 0.7 nm, respectively. The grids appeared homogeneous, as the uncertainty was relatively small for these nanometer features. The second path led to wider and shorter grids than those of the first pass. These observations can be rationalized by the higher degree of droplets spreading during the printing of the third-layer grids. The apex of the probe due to the printing of two layered grids was coated by the lipid molecules and as such increased the transient droplet spreading during the delivery. This explanation was confirmed by printing a linear feature at the end of the experiment and comparing its width with the first grid layer. The feature width exhibit an increase in comparison to that using the fresh probe.

Under the same design, the grid width and height could easily be tuned by changing the moving speed of the probe. For example, taller and wider cross-grids were produced when the speed was reduced from 150 to 8 µm/s; the height for the single and double lines increased to 300 and 470 nm, and the width increased to 2.3 and 2.6 µm, respectively. This stacking grid series indicates that this approach enables the 3D nanoprinting of complex lipid structures with nanometer precision over a millimeter footprint.

### 3.4. Production of Complex 3D Lipid Structures 

**Figure 5:**
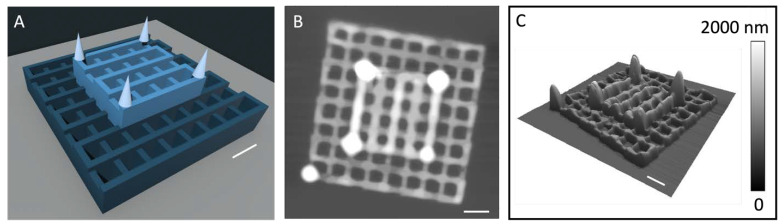
(**A**) Schematic diagram of a 3D structure used to 3D-print POPC. (**B**) Topographic AFM image of 3D POPC structure constructed following the design in (**A**). (**C**) A 3D display of the AFM image shown in (**B**). Scale bars = 5 µm.

Building on top of linear and stacking grid designs, our approach was also tested for more complex structures to demonstrate the genericness and spatial fidelity. One example of the designs that we tested is shown in Figure 5A: a large grid with a smaller grid stacking atop and in the middle area, and four cones as the top layer. The base grid had 2 line arrays with 9 lines each perpendicular to each other with periodicity of 4 µm, covering an area of 32 × 32 µm. Atop the base layer and in the middle region lay a 5 × 5 grid with the same design as that of the base. The lipid solution was produced by dissolving a POPC/NBD-PE mixture at 0.033 M in ethanol. An OTS/glass substrate was used as the support. To print the POPC grids, delivery pressure was kept at 20 mbar with a printing speed of 10 µm/s. The four lipid cones were produced by dispensing one droplet at a time with a contact duration of 10 ms under 10 mbar delivery pressure. The final 3D structure was characterized via AFM 4 days after curing and is shown in Figure 5B. Except for a bright feature at the corner of the base grid, due to the long “probe parking time” at the beginning and end, the 3D structure of POPC followed the design faithfully. The grids in the base and second layer measured 266 ± 33 and 206 ± 45 nm tall, respectively. The cones were 4.7 ± 0.5 µm in diameter and 1164 ± 279 nm in height at the base. A similar high fidelity was achieved for various other designs, and the outcomes demonstrate the reliability and high spatial precision of our approach.

### 3.5. Production of Organizational Chiral Lipid Structures 

**Figure 6:**
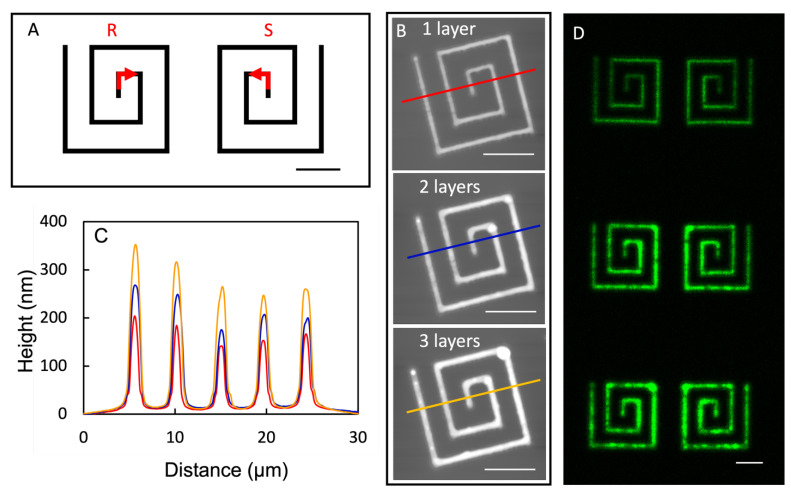
(**A**) A schematic diagram of the designed pair of R and S square spirals. (**B**) Topographic AFM images of the chiral POPC structures following the design of the R-structure in (**A**) with increasing height from top to bottom: 1, 2, and 3 passes, respectively. (**C**) Three cursor profiles indicated in (**B**). (**D**) Laser scanning confocal images acquired 10 days after the delivery for the structures shown in (**B**) (left) and their counterpart S-structures (right). All scale bars = 10 µm.

Organizational chirality has gained interest and attention from the fields of chemistry and bioengineering [54,55,56,57,58]. The current means to produce most organizational chiral structures relies on self-assembly [59,60,61] that produces chiral domains with broad size distribution, and the entire surface contains a racemic mixture of chiral domains [62,63]. With the demonstrated 3D nanoprinting capability, we should be able to, in principle, produce 3D lipid structures with the designed chirality at the designated locations. Under similar conditions as those in the previous section, the square spirals of POPC were designed (Figure 6A) and successfully printed. as shown in Figure 6B,C. In Figure 6B (top), a right-handed R-squared spiral is shown whose height and width (FWHM) were 169 ± 25 and 860 ± 51 nm, respectively. More printing passes enabled us to build up the height of these R-spirals to 220 ± 38 and 288 ± 44 nm with an additional 1 and 2 passes, respectively, as shown in Figure 6C. Its mirror image, S-square spirals, could also be produced by following the design shown on the right of Figure 6D. The successful production of organizational chiral structures opens opportunities for producing advanced chiral devices.

## 4. Conclusions

Combining atomic force microscopy (AFM) with microfluidic delivery probes, this work was utilized to produce three-dimensional (3D) lipid structures following a custom design. While AFM is well-known for its spatial precision in imaging and nanolithography in 2D, the development of AFM-based nanotechnology into 3D nanoprinting requires overcoming the technical challenges of controlling material delivery and interlayer registry. This work demonstrated the concept of the 3D nanoprinting of amphiphilic lipid molecules such as 1-palmitoyl-2-oleoyl-sn-glycero-3-phosphocholine (POPC). Various formulations of POPC solutions were tested to achieve point, line, and layer-by-layer delivery. The designed structures were nanometer thick disks, long linear spherical caps, stacking grids, and organizational chiral structures. The POPC molecules formed stacking bilayers in these constructions from our high-resolution structural characterizations. The printing reached nanometer spatial precision over a range of 0.5 mm. Work is in progress to achieve the further miniaturization of individual features and broadening the materials to other lipid molecules. The outcomes revealed the promising potential of our technology and methodology in the production of 3D structures from nanometer to continuum, opening opportunities in the biomaterial sciences and engineering, such as the production of 3D nanodevices, chiral nanosensors, and scaffolds for tissue engineering and regeneration.

## Data Availability

Not applicable.
